# Effect of Dietary Grape Pomace on Fattening Rabbit Performance, Fatty Acid Composition, and Shelf Life of Meat

**DOI:** 10.3390/antiox10050795

**Published:** 2021-05-17

**Authors:** Mohamed D. Bouzaida, Virginia C. Resconi, David Gimeno, Jakeline V. Romero, Juan B. Calanche, Marta Barahona, José L. Olleta, Gustavo A. María

**Affiliations:** Departamento de Producción Animal y Ciencia de los Alimentos, Facultad de Veterinaria, Instituto Agroalimentario de Aragón IA2, Universidad de Zaragoza—CITA, 50013 Zaragoza, Spain; bouzaidadhia@gmail.com (M.D.B.); dgimeno@unizar.es (D.G.); jakromero@unizar.es (J.V.R.); calanche@unizar.es (J.B.C.); martabm@unizar.es (M.B.); olleta@unizar.es (J.L.O.)

**Keywords:** animal feeding, grape by-product, meat quality, lipid oxidation, antioxidant properties

## Abstract

The use of agroindustry by-products in animal diets allows the use of residues that are not fit for human consumption. In this study, it was investigated whether fattening commercial rabbits during 30 days with a non-medicated feed, with 20% addition of grape pomace (GPD), affected production traits and the fatty acid composition, antioxidants properties, and the shelf life of the meat compared to a conventional strategy (CON). Furthermore, it was tested, by chromatographic analysis, whether this alternative diet allowed the transfer of phenolic compounds to the meat. Thirty-six weaned rabbits were allotted to the two treatments. In each treatment, 18 rabbits were fattened in three indoor cages, each housing three males and three female rabbits. No significant differences were found in live weights (*p* > 0.05), but the feed conversion rate and carcass weight and yield were found to be impaired in the GPD group (*p* ≤ 0.05). The GPD group had a higher intramuscular fat percentage (2.01 vs. 1.54), improved polyunsaturated/saturated fatty acids ratio (0.75 vs. 0.66), and better atherogenicity (0.71 vs. 0.83) and thrombogenicity (1.14 vs. 1.24) indexes, while the *n*-6*/n*-3 ratio was higher (25.4 vs. 20.3). Total volatile basic nitrogen in meat was lower in the GPD group (*p* = 0.01), suggesting a delayed spoilage. However, no improvements in total phenolic content, antioxidant capacity, reducing power, and lipid oxidation (*p* > 0.05) were found in the meat. Even though the GPD pellets offered to the animals had several grape-derived phenolic compounds, and higher antioxidant properties compared to the CON diet, none of the phenolic compounds detected in feeds were detected in the meat samples.

## 1. Introduction

Rabbit meat is traditionally part of a healthy Mediterranean diet and its nutritional characteristics, such as a low fat and cholesterol content and a favourable fatty acid profile, etc., [1], are widely recognised. The high percentage of polyunsaturated fatty acids (PUFA), including long chain *n*-3 fatty acids, means that this meat is valued in terms of human health, but at the same time this results in a greater proneness to lipid oxidation, reducing its shelf life [2,3]. Previous studies have shown that animal feeds can affect fatty acid composition, shelf life, and the amount of bioactive compounds in meat, and that by including certain ingredients, the potential of rabbit meat as a “functional food” can be capitalised [1]. In this respect, incorporating in animal diets by-products of industrial fruit and vegetable processes [4,5,6] is a well-accepted strategy due to its perceived naturalness and to the environmental benefits it affords by reducing waste.

During industrial winemaking processes large amounts of pomace are produced (mainly grape skins, pulp, seeds, and any unremoved stems), which represent almost 25% of the contents of grapes used for wine production [7]. This by-product has phenolic compounds with antimicrobial and antioxidant properties that can be used in animal feed, with promising results in live animals (health, productivity) and in the shelf life of their meat [8]. Additionally, grape pomace provides other secondary plant metabolites, such as tannins and lignin, and linoleic fatty acid, which could affect feed digestibility or change the fatty acid profile of the meat. In a previous study, grape pomace showed a slight reduction in protein and energy digestibility, as well as a lower feed efficiency, but it was concluded that this by-product could partially replace alfalfa hay in growing rabbits’ diets (from 100 to 300 g/kg total weight), depending on the availability and economic value of these two ingredients [4]. In rabbit bucks, including up to 20% of grape pomace in their diet improved semen quantity and quality, and also increased their antioxidant capacity without affecting body weight gain [9]. However, to the best of our knowledge, the effect of grape pomace in fattening rabbits’ diets on the quality of their meat has not been assessed hitherto. Based on the aforementioned previous studies, we expect that the inclusion of 20% of grape pomace in diets will not impair animals’ performance, but it could improve the shelf life of meat if the phenolic compounds from the by-product are transferred to the meat. Interesting findings have been observed in other species that could be applicable to rabbits, such as the inhibition of pathogenic bacteria in the gut in piglets [10], methane reduction in dairy cows [11], and an increase of polyunsaturated fatty acids and reduction in oxidation in the meat of chickens [12,13], pigs [14], and lambs [15].

Furthermore, in line with the European Union tendency, a specific Spanish national strategy has been developed to minimise the use of medical products in animal production. In this respect, the use of by-products rich in polyphenols can help produce food without using medicated feeds in rabbit diets, thus improving the image of the sector vis-a-vis consumers by presenting a more sustainable product.

The objective of this study was, therefore, to investigate the effect of including 20% of grape pomace by-product in non-medicated fattening rabbit diets on animal performance, and on the fatty acid composition, the phenolic profile, and shelf life of their meat, in comparison to a medicated commercial diet.

## 2. Materials and Methods

### 2.1. Animals and Experimental Design

The study that was performed at the Animal Experimentation Service of the University of Zaragoza, Spain (latitude 41°41′ N). The care and use of animals were performed in accordance with the Spanish Policy for Animal Protection RD53/2013, which meets the European Union Directive 2010/63 on the protection of animals used for experimental and other scientific purposes. Thirty-six New Zealand white rabbits were weaned at 35 days of age and allotted to two treatments, with the animals in each group balanced according to initial live weight. Each treatment consisted of three cages housing six rabbits each, balanced by sex (three females and three males), making a total of 18 animals per treatment. Treatments consisted of two ad libitum diets during 30 days:

- CON: A medicated diet (replaced by a withdrawal feed 1 week before slaughter).
- GPD: A non-medicated control diet supplemented with 20% grape pomace.

The commercial pellets contained alfalfa flour, vetch, fescue, ray-grass, barley, sunflower seed meal, corn gluten feed, soybean hulls, palm kernel pressure cake, sugar cane molasses, wheat bran, calcium carbonate, palm vegetable oil, sodium chloride, vitamins, and minerals. The CON diet had robenidine hydrochloride as the coccidiostat, unlike the withdrawal feed which was a non-medicated control diet without coccidiostat. Grape pomace from Granache grapes was supplied by a family winery from Cariñena (Aragón, Spain). The GPD was the result of mixing eight parts of ‘withdrawal feed’ and two parts of the grape pomace by-product. The withdrawal pellets and the by-product ground and new pellets were produced at the Animal Experimentation Service of the University of Zaragoza. Diet samples were taken during the experiment to analyse the chemical composition, total phenolic content, and antioxidant capacity (Table 1). The housing conditions established were an ambient temperature of 20 °C, 16L:8D lighting schedule, and free access to drinking water. Animals were fattened in traditional, flat-deck type cages (73 cm long × 47 cm wide × 30 cm high; 572 cm^2^/head). Feed consumption and rabbit weight were recorded weekly and at the end of the fattening period, as group data of the six animals per cage. The feed conversion ratio was calculated by dividing the feed consumed (kg) per kg of live weight gained.

### 2.2. Slaughtering and Meat Sampling

At 65 days of age, 18 rabbits per group were stunned and slaughtered without fasting, at a commercial slaughterhouse located at 28 km. After slaughter, carcasses (excluding all viscera and the distal parts of the tail and fore and hind legs, but including the head) were immediately transported to the Animal Production Unit Laboratory of the University of Zaragoza, where carcass weights were recorded. Carcass yield was obtained for the group of rabbits in each cage, calculated as the ratio between carcass weight and live weight at slaughter. Carcasses were refrigerated at 4 °C for 24 h and left hind leg samples were obtained, vacuum-packed, and stored at −20 °C until analysis. Before analysing, samples were thawed at 4 °C for 24 h, the meat was removed from the bone and minced to assess pH, thiobarbituric acid reactive substances (TBARs), and total volatile basic nitrogen (TVB-N) at 0, 4, and 6 days of storage at 4 °C, in overwrap. At day 0, samples of hind leg were freeze-dried to analyse the total phenolic content, antioxidant capacity, reducing power, and phenolic compounds. The fat percentage and fatty acid composition of the meat was analysed in a cranial section of the right *Longissimus dorsi* muscles, which were minced and freeze-dried.

### 2.3. Chemical Composition of the Diets, Grape Pomace, and Fat Percentage in Meat

Analyses of diet composition, grape pomace, and fat percentage of the meat were conducted at the Agrifood Research and Technology Centre of Aragón (CITA, Zaragoza, Spain) at the Physical-Chemical and Instrumental Analysis Laboratory. Feedstuff samples were oven-dried to determine the dry matter content. Crude protein and ether extract were determined according to the procedures of the Association of Official Analytical Chemists (AOAC) [16]. Neutral detergent fibre, acid detergent fibre, and acid detergent lignin analyses were carried out using the sequential procedure of Van Soest et al. [17], with the Ankom fibre analyser (Model 200/220, Ankom Technology, Gomensoro, Madrid, Spain). The fat percentage in freeze-dried, minced *Longissimus dorsi* muscle was also analysed using the aforementioned methodology and was expressed as a percentage of fresh meat.

### 2.4. Fatty Acid Composition of Diets and Rabbit Meat

Fatty acid profiling of freeze-dried diets and *Longissimus dorsi* muscle samples were conducted according to the adapted methodology of Lee et al. [18] at the Institute of Food Science, Technology and Nutrition (ICTAN) in Madrid. Samples of 0.1 g were weighed and placed in an ultrasound cleaned (USC) culture tube. Two mL of 0.5 M sodium methoxide in methanol and 1 mL hexane containing 1 mg/mL C13:0, as an internal standard, were added and then heated for 15 min at 50 °C. Acetyl chloride in methanol (1:10; *v*/*v*; 2 mL) was added before mixing thoroughly and heating for 1 h at 60 °C. Three mL of hexane, 1 mL of deionised water, and 0.2 g of anhydrous sodium sulphate were added, mixed, and centrifuged at 4 °C for 5 min at 1500 rpm. The organic solvent top layer was pipetted into a vial to be used for gas chromatography (GC) analysis. Fatty acid methyl esters (FAME) were assayed by gas chromatography with a flame ionization detector (Agilent 7820A), using a column (60 m × 0.25 mm × 0.25 μm, Agilent HP-23) with split injection, 1 μL (40:1), and helium at a constant flow of 1 mL/min, as the carrier gas. The detector temperature was set at 260 °C and the injector oven temperature at 250 °C. The initial temperature of the oven was 100 °C (held for 2 min) and then increased by 8 °C/min to 145 °C. This temperature was held for 20 min and then increased by 5 °C/min to 195 °C and held for 5 min. Afterwards, the temperature was increased by 5 °C/min to 215 °C and held for 5 min, and again increased by 5 °C/min to 230 °C and held for 5 min. The total analysis time was 59.6 min. Identification of fatty acids and their response factors was aided by the use of a reference standard (FAME 37 Supelco Ref CRM47885 + PUFA No. 2 Animal Source Ref 47015-U Sigma + PUFA No. 3 Menhaden oil Ref 47085-U) and quantified using the internal standard (C13:0).

Atherogenicity (AI) and thrombogenicity (TI) indices were calculated [19]: AI = (C12:0 + 4 × C14:0 + C16:0)/((MUFA + *n*-6) + *n*-3)); TI = (C14:0 + C16:0 + C18:0)/((0.5 × MUFA + 0.5 × *n*-6 + 3 × *n*-3) + (*n*-3/*n*-6)), together with the peroxidability index (PI), according to Arakawa and Sagai [20]: PI = (% monoenoic × 0.025) + (% dienoic × 1) + (% trienoic × 2) + (% tetraenoic × 4) + (% pentaenoic × 6) + (% hexaenoic × 8).

### 2.5. HPLC Analysis of Polyphenols

The analysis was carried out on freeze-dried diets and twelve meat samples (hind legs, 6 per group), with a Waters H-Class high-performance liquid chromatography (HPLC) system coupled to a quadrupole time-of-flight mass spectrometry (QTOF) equipped with an electrospray ionization (ESI) source (microTOF-Q, Bruker Daltonik), according to the modified method of Mena et al. [21]. The column used was an analytical HPLC column (ZORBAX Eclipse Plus C18, 50 mm × 2.1 mm i.d., 1.8 μm spherical particle size, Agilent). Autosampler and column temperatures were 10 and 30 °C, respectively. The injection volume was 5 μL and the flow rate was 0.4 mL/min. The mobile phase was built using two solvents: 0.1% formic acid in water (A), and 0.1% formic acid in acetonitrile (B). Initial conditions were 5% B for 1 min, then a 11-min linear gradient to 30% B followed by 1-min linear gradient to 80% B and maintaining this composition for 4 min. In order to regenerate the column a 5 min pre-run with 5% B was achieved before the next injection. Electrospray ionization collision-induced dissociation mass spectrometry ESI-CID-MS (QTOF) analysis was carried out in positive and negative ion mode, with capillary and endplate offset voltages of 4500 and −500 V in positive mode, and 4000 and −500 V in negative mode, using nitrogen as the collision gas. For MS/MS measurements, collision cell energies of 25 eV were used for positive and negative mode, with an isolation width for the precursor ion of 4 m/z units. To allow coupling with the HPLC system, the nebulizer gas (N_2_) pressure and the drying gas (N_2_) flow rate were 1.6 bar and 8.0 L min^−1^, respectively. The mass axis was calibrated externally and internally using Na-formate adducts. Bruker Daltonik o-TOF Control v.3.4, HyStar v.3.2 and Data Analysis v.4.2 software packages were used to control the MS (QTOF) apparatus, for the interface of the HPLC with the MS system and to process data, respectively. The two experimental diets, the wine industry grape pomace by-product and the meat sample extracts were ionized using the negative mode to detect phenolic acids, favan-3-ols, flavonols, and stilbenes, and the positive mode to detect anthocyanins. The resulting pseudomolecular ions (M − H)^−^ or (M + H)^+^ after fragmentation by collision-induced disassociation (CID) were separated by mass to charge ratio (*m*/*z*). Then, when enough collision energy was supplied, the precursor ions with a particular m/z were fragmented again and separated in a second stage of mass spectrometry (MS/MS). Phenolic compounds were tentatively identified based on the calculated mass of pseudomolecular ions, m/z of fragmented product ions, and retention times previously reported in the specific literature [22,23,24,25,26,27,28,29,30,31].

### 2.6. Total Phenolic Content, Antioxidant Capacity, and Reducing Power

The phenolic extract preparation was conducted in two steps. Samples of hexane-defatted and freeze-dried meat and animal diets (1.5 g) were homogenized in 10 mL of ethanol:water (80:20) using an Ultra-Turrax T25 (Janke & Kunkel, Staufen, Germany) for 60 s. The homogenate was then centrifuged at 4 °C for 15 min at 4000 rpm and the supernatant was collected. The extracted pellets were homogenised in 10 mL of acidified methanol 10% with HCl 1N using a vortex shaker for 60 s before being incubated for 60 min at 85 °C to extract hydrolysable polyphenolics following the procedure described by Hartzfeld et al. [32] and Lei et al. [33] with slight modifications. They were then centrifuged at 4000 rpm for 15 min at 4 °C and evaporated to dryness in a rotary evaporator at 40 °C before being reconstituted with the supernatant obtained in the previous step, resulting in a crude extract containing both free and hydrolysable phenolic fractions. Crude extracts were subjected to the solid phase extraction (SPE) treatment with the Oasis HLB cartridge (200 mg, 3 cc, 30 μm particle size) filtered through a 0.45 μm membrane filter and kept at −18 °C until analysis. The final supernatant obtained was used for the estimation of total phenolic content, 2,2-diphenyl-1-picrylhydrazyl (DPPH), ferric reducing antioxidant power (FRAP), and for polyphenolic compound profiling by HPLC-ESI-CID-MS (QTOF).

The total phenolic content (TPC) in meat and animal diets was determined using the Folin–Ciocalteu reagent following the modified method of Singleton et al. [34]. Folin–Ciocalteu was added (0.5 mL) to 0.5 mL final extract, followed by the addition of 0.5 mL sodium carbonate solution (7.5%) and 7 mL of distilled water. The reaction mixture was vortexed before being incubated for 1 h in the dark. The absorbance of phenolic content was measured spectrophotometrically with a UV-20 Onda Spectrophotometer at 760 nm. The quantification of phenolics was based on the standard curve generated with the use of gallic acid and expressed as mg of gallic acid equivalent/100 g dry weight.

The DPPH radical scavenging activity was estimated according to Llorach et al. [35]. Results were expressed as μmol of Trolox equivalents/100 g dry weight after measuring the absorbance of the DPPH radical-extract complex and the Trolox standard curve (0–60 μM) at 515 nm.

The FRAP assay was performed according to Thaipong et al. [36] with some modifications. The stock solutions included a 23 mM acetate buffer (3.13 g C_2_H_3_NaO_2_·3H_2_O and 16 mL acetic acid), pH 3.6, 10 mM TPTZ (2, 4, 6 tripyridyl-s-triazine) solution in 40 mM HCl, and 20 mM ferric chloride hexahydrate solution. The fresh working solution was prepared by mixing 25 mL acetate buffer, 2.5 mL TPTZ solution, and 2.5 mL ferric chloride hexahydrate solution and then warmed at 37 °C before using. Samples of the meat extract (120 μL) were allowed to react with 900 μL of the FRAP solution for 20 min in the dark condition. Readings of the coloured product [ferrous tripyridyltriazine complex] were then taken at 595 nm. The standard curve was linear between 50 and 1000 μM Trolox. Results were expressed in μmol of Trolox equivalents/100 g dry weight. Additional dilution was needed if the FRAP value measured was over the linear range of the standard curve.

### 2.7. Evaluation of pH

The pH was determined in minced left hind leg meat at 0, 4, and 6 days of refrigerated storage (4 °C) in overwrap using an XSPH7 + DHS portable pH-meter with a penetration probe.

### 2.8. Thiobarbituric Acid Reactive Substances Assay

Lipid oxidation was quantified using the thiobarbituric acid reactive substances (TBARs) assay according to Pfalzgraf et al. [37]. Meat samples weighing 10 g ± 0.02 were homogenised with 20 mL of trichloroacetic acid (10%) using an Ultra-Turrax T25 (Janke & Kunkel, Staufen, Germany). Samples were centrifuged (Gyrozen 1248R, Daejeon, Korea) at 4000 rpm for 30 min and the supernatants filtered through qualitative paper (F1093 grade; Chmlab, Barcelona, Spain). Two milliliters of the filtrates were taken in duplicates and mixed with 2 mL of thiobarbituric acid, homogenized, and incubated for 20 min in a water bath at 97 °C. Absorbance was measured at 532 nm. The results were expressed as mg of malondialdehyde (MDA)/kg of the sample, using a standard curve that covered the concentration range of 1 to 10 mM 1,1,3,3-tetramethoxypropane. Lipid oxidation assays were performed at 0, 4, and 6 days of storage at 4 °C, in overwrap samples.

### 2.9. Total Volatile Basic Nitrogen Assay

The total volatile basic nitrogen (TVB-N) assay was carried out according to the protocol described in the European Union Commission Regulation 2074/2005, Chapter III, “*Determination of the concentration of TVB-N in fish and fish products*” [38] with some modifications. A 2 g sample of homogenised minced rabbit meat was blended with 90 mL of 6% perchloric acid solution using an Ultraturrax (IKA, Staufen, Germany). The extract obtained was filtered, and 50 mL aliquots were dispensed into an apparatus for steam distillation. Analyses were run in triplicate. The 50 mL of filtrate was rendered alkaline with 20% hydroxide and distilled in a Udk 129 Kjeltec Distillation Unit (VELP Scientifica Srl, Usmate (MB)—Italy. The titration was carried out with 0.01 N HCl, and the results were reported as mg N_2_ non-protein/100 g fresh rabbit meat.

### 2.10. Statistical Analyses

Statistical analyses were performed using the SPSS software package (version 26.0). Analysis of variance was used to evaluate productive performance traits, fat percentage, fatty acid profile, total phenolic content, and DPPH and FRAP of rabbit meat, according to the dietary treatment (CON vs. GPD) as the source of variation. TBARs, TVB-N, and pH data on meat were processed using a general linear model, with diet, days of storage and their interaction as fixed factors. Marginal mean values and standard error were reported. A probability value of *p* ≤ 0.05 was considered statistically significant. Tukey’s multiple range test was used to determine whether there were significant differences between the mean values of storage days.

## 3. Results and Discussion

### 3.1. Productive Performance

Performance traits for rabbits fattened with grape pomace supplementation and a commercial diet are presented in Table 2. No significant differences between the two diets were found for periodically assessed live weight (*p* > 0.05), but the feed conversion rate was higher and carcass weight and yield were lower for the grape pomace diet compared to the control diet (*p* ≤ 0.05).

Supporting our results, those of Motta-Ferreira et al. [4] showed a similar daily gain and increasing feed conversion rates, when alfalfa hay was replaced with grape pomace in increasing proportions (from 0 to 300 g/kg total weight). Likewise, Eid [9] showed similar weight gains in rabbit bucks of 6 months of age that were fed a basal diet or a diet supplemented with 10 or 20% grape pomace (both being isonitrogenous and isocaloric). The results of our study are probably related to the fact that grape pomace is a highly lignified source of fibre, which could increase the transit time of digesta and reduce nutrient digestibility [39], stimulating feed intake while achieving a similar growth rate. In a previous study, the increased levels of fat provided by grape pomace may encourage consumption [4], but in our study there was little difference in ether extract between the diets (Table 1).

Generally, a diet that enhances feed intake is assumed to improve carcass yield by reducing caecum content weight [40], however, and in line with our results, the study by Margüenda et al. [41] found lower carcass yields when rabbits were fed a high fibre straw diet compared to a low fibre straw diet, but no differences in digestive tract weight were observed. A recent study concludes that the effect of lignin rich diets on carcass yields is not consistent and that interaction factors should be considered [42], whereas the effect of high soluble fibre on reducing carcass yield is more reliable [43].

### 3.2. Fat Percentage and Fatty Acid Composition of the Meat

The fat percentage and the fatty acid composition of the intramuscular fat of rabbits that were fed the control diet (CON) or the alternative diet containing 20% grape pomace (GPD) is reported in Table 3. The inclusion of grape pomace increased the fat percentage (2.01 vs. 1.54) and modified the fatty acid composition of the *Longisimuss dorsi* (LD) muscle. In a previous study, grape pomace inclusion in fattening diets increased the body fat content of rabbits slaughtered at 2 kg live weight and the results were related to increased ether extract and reduced digestible protein to digestible energy ratio and the increased feed intake achieved with the grape by-product enriched diets [4], and all these might play a part in explaining the differences in our study, as well.

A higher percentage of intramuscular fat suggests a higher proportion of triacylglycerol and a lower proportion of phospholipids, which in rabbits means increased C14:0 and C16:0 and monounsaturated fatty acids (MUFA), and decreased polyunsaturated fatty acids (PUFA) (except C18:3 *n*-3) and C18:0 fatty acids percentages [44], but all these changes were not evident in the GPD group (Table 3). Therefore, it was diet composition more than fat percentage that was responsible for the differences found in the fatty acid profile of LD samples, as was also observed in a study of rabbit diets supplemented with artichoke bracts [5]. The fatty acid composition of the diet is reflected directly in essential fatty acids, such as linoleic and linolenic fatty acids, but it could also affect the accumulation and synthesis of other fatty acids [45,46] in the animals.

The GPD group showed a lower percentage of saturated fatty acids (SFA), mainly due to the lower proportion of palmitic (C16:0) and myristic (C14:0) acids, and increased PUFA due to the higher proportion of linoleic acid, but a lower proportion of α-linolenic acid, leading to PUFA/SFA and *n*-6/*n*-3 ratios that were higher in the alternative diet group than in the control group (Table 3). These results are in agreement with those reported by Bennato et al. [13] in poultry meat that showed that diets containing at least 5% of grape pomace by-product induced an increase in the concentration of linoleic acid and a decrease in total SFA, increasing the PUFA/SFA ratio.

Our findings specifically suggest that GPD plays a positive role in increasing the PUFA/SFA ratio but involves an undesirable rise of the *n*-6/*n*-3 ratio compared to the control diet, as a consequence of the high linoleic acid percentage. Both treatments achieve the level recommended by the Department of Health [47] for the PUFA/SFA ratio, but not the *n*-6/*n*-3 ratio, these being >0.4 and <4, respectively.

Other nutritional indices are the atherogenic index (AI) and the thrombogenic index (TI), which, the higher they are the higher the risk is of developing atherosclerosis and platelet aggregation in humans [19]. In our study, these two indices were lower in the meat from the experimental diet in comparison with the control diet, as is the case in previous studies with other polyphenol-rich feed ingredients, since, additionally, they provide fatty acids that improve the two aforementioned indices [6,48]. The single fatty acids that played a greater role in this improvement were the reduction of C14:0 and C16:0 and the increase of C18:6 *n*-6 in the meat of the GPD group.

### 3.3. Identification of Phenolic Compounds

Table 4 shows that among the different feed samples 42 phenolic compounds were identified, 38 of them appeared in the grape pomace by-product of which 14 compounds belonged to the flavanol group, mainly procyanidins and their derivatives and catechin 3-*O*-gallate, six compounds correspond to the flavonols group, seven were anthocyanins, eight resveratrol derivatives and ellagic acid belonging to phenolic acids. The grape pomace by-product compounds identified were also detected in previous studies of grape derivatives [22,23,24,25,26,27,28,29,30,31,49].

As was expected, in the grape pomace diet most of the phenolic compounds present in the by-product were found, with the exception of procyanidin A dimer digallate, syringetin 3-*O*-hexoside, procyanidin A dimer gallate, resveratrol tetramer, malvidin 3-*O*-galactoside, malvidin 3-*O*-hexoside-acetaldehyde, delphinidin 3-*O*-hexuronide, and peonidin 3-*O*-(6′′-p-coumaroyl)-hexoside.

On the other hand, three compounds: Chlorogenic acid, chlorogenic acid hexoside, and malvidin 3-*O*-hexoside derivative were detected in the two diets, but not in the grape pomace by-product, whereas other six compounds were detected in all the feed samples: Procyanidin B dimer (R_t_ 2.7), myricetin O-dihexoside, ellagic acid, quercetin 3-*O*-hexuronide, quercetin, and kaempferol. However, as shown in Figure S1, these last two compounds, quercetin (peak 28) and kaempferol (peak 33), were found in higher concentrations in the GPD and even more so in the grape by-product. These two flavonols have also been reported in various wine grape varieties [50]. Finally, quercetin 3-*O*-galactoside was only detected in the control diet (Table 4).

None of the phenolic compounds detected in feeds reported in Table 4 were detected in the meat samples. Few studies have analysed dietary phenolic compounds in meat. For example, in the study by Moniño et al. [51] eleven phenolic compounds detected in pellets fed to ewes were also detected in the meat of their suckling lambs without modification. In contrast, other studies have shown that dietary phenolic compounds can be transformed, degraded, excreted or retained in other tissues, such as the liver [52,53,54], and therefore, they are not detected in meat.

### 3.4. Total Phenolic Content, Antioxidant Capacity, and Reducing Power

The total phenolic content, antioxidant capacity measured as DPPH (2,2-diphenyl-1 picrylhydrazyl) free radical scavenging activity, and reducing power measured by the FRAP method (ferric-reducing ability) in hind leg rabbit meat were not affected significantly (*p* ≤ 0.05) by dietary grape pomace supplementation (Table 5). Although it is difficult to compare results from different studies due to divergences in the extraction and analytical methods, our results are in agreement with those of Mancini et al. [55] and North et al. [56], in that no effect on antioxidant properties was observed in the meat of rabbits offered diets supplemented with ingredients rich in polyphenols (4 or 8 g of *Zingiber officinale Roscoe* (ginger) powder in 100 g of feed or with 2 g quercetin/kg feed, respectively). However, our results contrast to those of Perna et al. [6] and Menchetti et al. [57], who reported greater total phenolic content and antioxidant capacity in the meat of rabbits that were fed diets including cauliflower and goji berries, respectively, compared to the control groups. Regarding grape pomace, Goñi et al. [12] showed that a dietary inclusion rate of up to 30 g/kg increased antioxidant activity in diet, excreta, and meat in broilers. Zhao et al. [15] showed that the total antioxidant capacity, glutathione peroxidase 4, and superoxide dismutase activity increased in ram lambs fed with diets containing wine grape pomace (with levels of 0%, 5%, and 10%).

The DPPH and FRAP levels of the grape pomace by-product used in our study are similar to the levels reported by Rockenbach et al. [50], but the TPC is lower than the mean values they reported (from 3200 to 7400 mg gallic acid equivalent per 100 g of dry weight, depending on the grape varieties). By including 20% of this by-product, TPC values increased 119%, DPPH 300%, and FRAP 98% in the pellet diets of the present study (Table 1). Despite the differences in the two diets (control vs. grape pomace), no significant effect was found on the aforementioned parameters (Table 5), either in meat or lipid oxidation (TBARs, Table 6). The lack of effect in the GPD group may be due to the loss of antioxidant properties after digestion or to a low/null deposition of antioxidants in the meat. Dabbou et al. [48] found an increase in antioxidant activity in the kidney and liver of growing rabbits, but a similar degree of lipid oxidation in the meat when supplementing diets with bilberry pomace vs. control diets. With grape pomace added in feedstuffs, Eid [9] found antioxidant effects of this by-product in rabbit buck semen, which means that the antioxidant properties can be transferred to the animal, but maybe not to a similar extent to the meat.

### 3.5. Evaluation of pH

Regarding the pH in minced meat from the hind leg, our data (Table 6) are within the range of what other studies have reported for that cut [58,59,60]. No effect of diet or of storage time was found (Table 6). These results are in agreement with the review of Hulot and Ouhayoun [60] who stated that diet usually does not affect the pH, especially when the growth rate is not affected. Likewise, Perna et al. [6] and North et al. [56] did not find any significant influence of dietary cauliflower and quercetin on rabbit meat pH. With regards to the evolution of pH during refrigerated storage, our results are in contrast to general findings [56,60,61] which report an increase in pH over time due to protein deamination.

### 3.6. Lipid Oxidation

Although the PUFA/SFA ratio was higher in meat samples from GPD, the peroxidability index (PI) showed the same susceptibility to lipid oxidation between dietary treatments (Table 3). In addition, the lack of any difference in the antioxidant properties of the meat from the two treatments (Table 5), shows that the malondialdehyde concentration found in meat from the two diets is similar (Table 6). However, as expected, TBARs levels increased (*p* ≤ 0.05) in minced hind leg samples stored at 4 °C, from 0.10 mg MDA/kg on the initial day (d0) to 0.42 on the sixth day (d6). In previous studies, dietary grape pomace supplementation was able to reduce TBARs in broiler meat and patties, this inhibition being clearer the longer the storage days [12,62].

### 3.7. Total Volatile Basic Nitrogen

The total volatile basic nitrogen indicates protein and amine degradation due to microbial activity or to endogenous enzymatic action and is generally used to estimate the shelf life of fish and meat [63]. TVB-N was lower in the GPD group (Table 6), suggesting that 20% grape pomace in diets may delay spoilage in rabbit meat. Phenolic compounds in grape by-products have well-known antimicrobial effects [8,64], but in the present study we could not identify them in the meat samples, making further studies necessary. The amount of TVB-N content increased significantly during refrigerated storage at 4 °C, going from 29.66 on the initial day (d0) to 43.00 mg/100 g of meat on the sixth day (d6) (Table 6). Other studies have demonstrated that microorganisms in chicken meat raise the proteolysis degree and lead to increases in the TVB-N value [65,66]. In meat, unlike fish, there are no official TVB-N reference values or limits. In fresh rabbit meat, Castrica et al. [67] and Lan et al. [68] reported TVB-N mean values of 15.4 and 8.6 mg/100 g at day 0 to 16.3 and 23.4 mg/100 g at day 10 of refrigerated storage, respectively. Our results were higher than the findings of these previous studies but in the present experiment thawed rabbit meat was minced and stored in refrigerated conditions. Frozen storage significantly worsens protein degradation, increasing basic volatile nitrogen due to protein deamination [69].

## 4. Conclusions

Adding 20% grape pomace to the diet did not affect the live weights of rabbits during the fattening period, however it reduced the feed conversion rate and carcass weight and yield. The resultant meat had a higher intramuscular fat percentage and a beneficial fatty acid composition of the *Longissimus dorsi* with respect to the polyunsaturated/saturated fatty acid ratio and the lower atherogenicity and thrombogenicity risks. However, the *n*-6/*n*-3 ratio was found to be unfavourable. Several phenolic compounds, such as quercetin, kaempferol, myricetin, and resveratrol derivatives, were highlighted in the diet with added grape pomace, likewise, the total phenolic content, antioxidant capacity, and reducing power were higher compared to the control diet. However, these phenolic compounds were not detected in the meat, and the two treatments, showed similar levels of antioxidant capacity and reducing power. Regarding meat shelf life, dietary inclusion of grape pomace apparently decreased protein spoilage of rabbit meat, but no effect on lipid oxidation was found in minced hind leg meat stored up to 6 days.

Finally, the present study shows that grape pomace is a suitable ingredient for use in fattening rabbit diets and could help reduce the use of medical products in animal nutrition and further the sustainability of farming systems, however there are other aspects such as feed costs that must be taken into consideration.

## Figures and Tables

**Table 1:** Chemical composition, fatty acid composition, total phenolic content, antioxidant capacity (DPPH), and reducing power (FRAP) of the two experimental diets and the wine industry grape pomace by-product.

	Control Diet	Grape Pomace Diet	Grape Pomace by Product
Chemical composition (%)			
Dry matter	91.54	91.02	88.52
Ether extract	4.61	4.97	7.01
Crude protein	14.08	14.16	11.66
Neutral detergent fibre	53.46	47.7	63.44
Acid detergent fibre	33.60	35.04	57.93
Acid detergent lignin	7.84	12.66	41.93
Fatty acid composition (% of total fatty acids)			
C12:0	12.33	5.90	0.27
C14:0	4.60	2.51	0.51
C16:0	18.65	18.83	16.95
C18:0	2.47	2.95	5.19
C16:1 *n*-7	0.24	0.33	1.39
C18:1 *n*-7c	0.71	0.85	1.05
C18:1 *n*-9c	23.98	31.36	19.26
C18:2 *n*-6c	31.40	33.54	49.40
C18:3 *n*-3	3.43	2.01	2.99
C22:6 *n*-3	0.28	0.21	0.25
Saturated fatty acids	39.57	31.25	25.66
Monounsaturated fatty acids	25.33	32.99	21.69
Polyunsaturated fatty acids	35.11	35.76	52.64
*n*-3 Polyunsaturated fatty acids	3.71	2.22	3.24
*n*-6 Polyunsaturated fatty acids	31.40	33.54	49.40
Total phenolic content (mg GAE/100 g d.w)	224	491	1737
DPPH (μmol Trolox equivalents/100 g d.w)	3253	13,032	58,764
FRAP (μmol Trolox equivalents/100 g d.w)	1843	3657	14,737

GAE: Gallic acid equivalent; DPPH: Free radical 2.2-diphenyl-1-picrylhydrazyl; FRAP: Ferric reducing antioxidant power; d.w: Dry weight.

**Table 2:** Marginal means and SE for productive traits of rabbits fattened with a commercial (medicated plus withdrawal feeds) or an alternative (unmedicated feed with 20% addition of grape pomace) feeding regime.

Variable	Control Diet	Grape Pomace Diet	SE	*p*-Value
Initial age (days)	35	35		
Days of fattening	30	30		
Final age (days)	65	65		
Initial live weight (kg)	7.40	6.36	0.41	0.146
Live weight (kg) week 1	9.72	8.48	0.47	0.140
Live weight (kg) week 2	11.93	10.71	0.51	0.167
Live weight (kg) week 3	14.26	12.90	0.55	0.157
Live weight (kg) at slaughter	16.50	14.83	0.58	0.115
Feed conversion *	3.38	3.66	0.10	0.001
Carcass weight (kg)	10.22	8.97	0.34	0.050
Carcass yield (%)	61.97	60.18	0.35	0.021

Group data from six animals per cage (three cages per treatment). * Diet (kg) per live weight gain (kg). SE: Standard error.

**Table 3:** Marginal means and SE for fat percentage, fatty acid profile (% of total fatty acids), and fatty acid ratios and nutritional indices of the *Longissimus dorsi* muscle of rabbits fattened with a commercial (medicated plus withdrawal feeds) or an alternative (unmedicated feed with 20% addition of grape pomace) feeding regime.

	Control Diet	Grape Pomace Diet	SE	*p*-Value
Fat	1.54	2.01	0.14	0.026
SFA	42.4	39.8	0.29	≤0.001
C10:0	0.26	0.19	0.02	0.010
C12:0	1.71	1.38	0.05	≤0.001
C14:0	4.17	3.47	0.12	≤0.001
C15:0	0.47	0.44	0.01	0.022
C16:0	28.2	26.8	0.30	0.002
C17:0	0.44	0.45	0.01	0.439
C18:0	6.66	6.71	0.14	0.809
C21:0	0.50	0.42	0.03	0.027
C24:0	0.48	0.36	0.03	0.007
MUFA	29.3	30.0	0.43	0.252
C14:1 *n-*5	0.22	0.16	0.02	0.011
C16:1 *n-*7	2.55	1.99	0.17	0.024
C18:1 *n-*7c	1.29	1.22	0.04	0.184
C18:1 *n-*9c	25.0	26.4	0.35	0.008
C20:1 *n*-9	0.28	0.27	0.01	0.911
PUFA	27.8	29.8	0.65	0.037
C16:3 *n*-4	0.57	0.54	0.04	0.519
C18:2 *n-*6c	20.7	23.5	0.33	≤0.001
C18:3 *n-*3	1.12	0.96	0.03	0.001
C20:4 *n-*6	4.11	3.60	0.28	0.210
C22:4 *n-*6	1.15	1.08	0.08	0.540
C22:6 *n-*3	0.17	0.15	0.01	0.307
*n-*3 PUFA	1.29	1.12	0.02	≤0.001
*n-*6 PUFA	26.0	28.2	0.61	0.016
*n*-6*/n*-3	20.3	25.4	0.73	≤0.001
PUFA/SFA	0.66	0.75	0.02	0.003
PI	48.2	48.0	1.82	0.926
AI	0.83	0.71	0.02	≤0.001
TI	1.24	1.14	0.01	≤0.001

SFA: Sum of saturated fatty acids; MUFA: Sum of monounsaturated fatty acids; PUFA: Sum of polyunsaturated fatty acids; PI: Peroxidability index; AI: Atherogenicity index; TI: Thrombogenicity index. SE: Standard error.

**Table 4:** HPLC-ESI-QTOF profile in negative and positive ionization mode (IM) of phenolic compounds identified in the control diet (CON), the grape pomace diet (GPD), the by-product grape pomace (GP), and in meat from the two groups.

N°	R_t_	Compound Name	IM	MS (*m*/*z*)	MS/MS (*m*/*z*)	CON	GPD	GP	Meat
1	0.3	Procyanidin trimer gallate	(M − H)^−^	1017.19	865, 729, 577, 575, 441, 125, 407, 289, 169	−	+ *	+	−
2	0.3	Chlorogenic acid	(M − H)^−^	353.0863	191	+	+	-	−
3	0.3	Cyanidin 3,5-*O*-diglucoside	(M + H)^+^	579.1408	287	-	+	+	−
4	1.0	Procyanidin A dimer	(M − H)^−^	575.1178	125, 113, 175, 289	-	-	+	−
5	2.7	Procyanidin B dimer	(M − H)^−^	577.1297	175, 113, 407	+	+	+	−
6	4.2	Procyanidin B trimer	(M − H)^−^	865.1949	577, 407, 125, 289	−	+ *	+	−
7	5.3	Malvidin 3-*O*-galactoside	(M + H)^+^	493.1349		−	-	+	−
8	5.4	Procyanidin B trimer	(M − H)^−^	865.1920	729, 575, 113, 175, 289, 287, 125	−	+	+	−
9	5.7	Procyanidin B dimer gallate	(M − H)^−^	729.1438	577, 175, 113, 425, 407, 289	−	+ *	+	−
10	5.8	Procyanidin A dimer gallate	(M − H)^−^	729.1326	575, 175, 113, 125, 289, 449	−	+ *	+	−
11	6.1	Procyanidin A dimer	(M − H)^−^	575.1172	175, 113, 289	−	+	+	−
12	6.6	Catechin 3-*O*-gallate	(M − H)^−^	441.0783	169, 125, 289	−	+	+	−
13	6.7	Myricetin *O*-dihexoside	(M − H)^−^	641.1413	297, 191, 125	+	+	+	−
14	6.8	Procyanidin A dimer digallate	(M − H)^−^	881.1572		−	−	+	−
15	6.8	Malvidin 3-*O*-hexoside-acetaldehyde	(M + H)^+^	517.1329		−	−	+	−
16	7.0	Ellagic acid	(M − H)^−^	300.9990		+	+	+	−
17	7.1	Delphinidin 3-*O*-hexuronide	(M − H)^−^	479.0810		−	−	+	−
18	7.2	Quercetin 3-*O*-hexuronide	(M − H)^−^	477.0645	301	+	+	+	−
19	7.2	Procyanidin A dimer	(M − H)^−^	575.1121	289, 125	−	+	+	−
20	8.1	Procyanidin A dimer	(M − H)^−^	575.1189	285, 125, 175, 113	−	+	+	−
21	8.4	Myricetin	(M − H)^−^	317.0322	151, 137	−	+	+	−
22	8.5	Syringetin 3-*O*-hexoside	(M − H)^−^	507.1135	344	−	-	+	−
23	8.6	Malvidin 3-*O*-hexoside derivative	(M + H)^+^	517.1296		+	+	-	−
24	8.7	Procyanidin A dimer gallate	(M − H)^−^	729.1408	407, 289	−	−	+	−
25	8.7	Chlorogenic acid hexoside	(M − H)^−^	515.1115	353, 179, 173	+	+	-	−
26	9.3	Malvidin 3-*O*-(6′′-p-coumaroyl)-glucoside	(M + H)^+^	639.1637		−	+	+	−
27	9.7	Malvidin 3-*O*-(6′′-p-coumaroyl)-exoside	(M + H)^+^	639.1674		−	+	+	−
28	10.2	Quercetin	(M − H)^−^	301.0382	151, 121, 179	+	+	+	−
29	10.6	Peonidin 3-*O*-(6′′-p-coumaroyl)-hexoside	(M + H)^+^	609.1559		−	−	+	−
30	11.2	Resveratrol dimer	(M − H)^−^	453.1291		−	−	+	−
31	11.4	Resveratrol tetramer	(M − H)^−^	905.2565	359, 451, 717	−	−	+	−
32	11.6	Resveratrol dimer	(M − H)^−^	453.1318		−	+	-	−
33	11.9	Kaempferol	(M − H)^−^	285.0422	136, 133, 273	+	+	+	−
34	12.0	Resveratrol dimer	(M − H)^−^	453.1309	369	−	+	+	−
35	12.3	Resveratrol tetramer	(M − H)^−^	905.2454		−	-	+	−
36	12.4	Resveratrol trimer	(M − H)^−^	679.1972		−	+	-	−
37	12.6	Resveratrol trimer	(M − H)^−^	679.1898		−	+	+	−
38	12.8	Resveratrol trimer	(M − H)^−^	679.1950	437	−	+	+	−
39	13.0	Resveratrol trimer	(M − H)^−^	679.1956		−	+	+	−
40	13.3	Resveratrol trimer	(M − H)^−^	679.1913		−	+	+	−
41	13.3	Quercetin 3-*O*-galactoside	(M − H)^−^	463.0915		+	−	−	−
42	15.9	Procyanidin B dimer	(M − H)^−^	577.3678		−	−	+	−

R_t_: Retention time (min), + detected compound and undetected compound, * appear as procyanidin B dimer with *m*/*z* 577.123 or procyanidin A dimer with *m*/*z* 575.1153.

**Table 5:** Marginal means and SE for total phenolic content (TPC), antioxidant capacity (DPPH), and reducing power (FRAP) of hind leg meat of rabbits fattened with a commercial (medicated plus withdrawal feeds) or an alternative (unmedicated feed with 20% addition of grape pomace) feeding regime.

	Control Diet	Grape Pomace Diet	SE	*p-*Value
TPC (mg gallic acid equivalent/100 g d.w.)	141.9	145.5	6.90	0.325
DPPH (μmol of Trolox equivalents/100 g d.w.)	93.95	98.09	2.93	0.720
FRAP (μmol of Trolox equivalents/100 g d.w.)	156.1	163.6	6.47	0.417

DPPH: Free radical 2,2-diphenyl-1-picrylhydrazyl; FRAP: Ferric reducing antioxidant power; d.w.: Dry weight.

**Table 6:** Marginal means and (SE) for pH, thiobarbituric acid reactive substance (TBARs), and total volatile basic nitrogen (TVB-N) of minced overwrapped hind leg meat stored for 6 days at 4 °C, from rabbits fattened with a commercial (medicated plus withdrawal feed) or an alternative (unmedicated feed with 20% addition of grape pomace) feeding regime.

Parameter	Storage	Diet			*p-*Value		
		Control	Grape Pomace	Mean	Diet	Storage	Diet X Storage
pH	*Day 0*	5.91	5.94	5.93 (0.01)			
	*Day 4*	5.92	5.99	5.96 (0.02)			
	*Day 6*	5.97	5.96	5.96 (0.01)			
	Mean	5.93 (0.01)	5.96 (0.01)		0.051	0.100	0.132
TBARs (mg MDA/kg)	*Day 0*	0.10	0.10	0.10b (0.05)			
	*Day 4*	0.31	0.35	0.33a (0.06)			
	*Day 6*	0.40	0.44	0.42a (0.05)			
	Mean	0.27 (0.04)	0.30 (0.04)		0.652	≤0.001	0.958
TVB-N (mg N/100 g)	*Day 0*	31.31	28.00	29.66c (1.02)			
	*Day 4*	35.45	33.36	34.41b (1.01)			
	*Day 6*	46.17	39.83	43.00a (1.71)			
	Mean	37.64 (1.05)	33.73 (1.06)		0.010	≤0.001	0.565

a, b, c Marginal means with different letters in the same column differ significantly (Tukey test). SE: Standard error.

## Data Availability

Data is contained within the article and supplementary material.

## Supplementary Materials

The following are available online at https://www.mdpi.com/article/10.3390/antiox10050795/s1. Figure S1: HPLC-ESI-QTOF chromatograms of phenolic compounds identified in the control diet (CON), the grape pomace diet (GPD), the by-product grape pomace (GP), and meat from the two groups (selected ion profiles in negative ionization).
