# Supplementary material for: Effect of Dietary Grape Pomace on Fattening Rabbit Performance, Fatty Acid Composition, and Shelf Life of Meat
Source: Antioxidants (Basel). 2021 May 17;10(5):795. doi: 10.3390/antiox10050795 (PMC8155864; doi:10.3390/antiox10050795)
Supplement: Supplementary file 1 [file antioxidants-10-00795-s001.zip › antioxidants-1210665-supplementary.pdf]

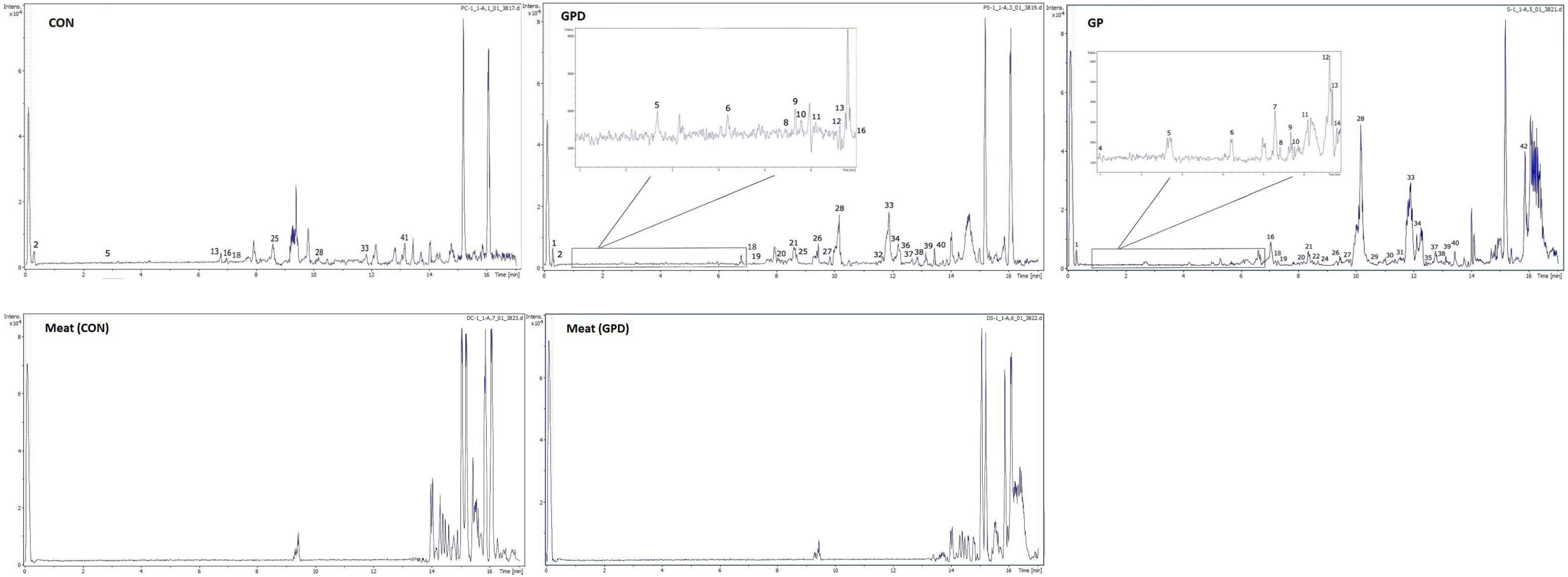

Figure S1. HPLC-ESI-QTOF chromatograms of phenolic compounds identified in the control diet (CON), the grape pomace diet (GPD), the by-product grape pomace (GP) and meat from the two groups (selected ion profiles in negative ionization)

Compounds identified: procyanidin trimer gallate (1), chlorogenic acid (2), procyanidin A dimer (4), procyanidin B dimer (5), procyanidin B trimer (6), malvidin 3-O-galactoside (7), procyanidin B trimer (8), procyanidin B dimer gallate (9), procyanidin A dimer gallate (10), procyanidin A dimer (11), catechin 3-O-gallate (12), myricetin O-dihexoside (13), procyanidin A dimer digallate (14), ellagic acid (16), quercetin 3-O-hexuronide (18), procyanidin A dimer (19), procyanidin A dimer (20), myricetin (21), syringetin 3-O-hexoside (22), procyanidin A dimer gallate (24), chlorogenic acid hexoside (25), malvidin 3-O-(6′′-p-coumaroyl)-glucoside (26), malvidin 3-O-(6′′-p-coumaroyl)-hexoside (27), quercetin (28), resveratrol dimer (30), resveratrol tetramer (31), resveratrol dimer (32), kaempferol (33), resveratrol dimer (34), resveratrol tetramer (35), resveratrol trimer (36), resveratrol trimer (37), resveratrol trimer (38), resveratrol trimer (39), resveratrol trimer (40), quercetin 3-O-galactoside (41) and procyanidin B dimer (42).
